# Efficient one step extraction process of Gramicidin S from *Aneurinibacillus aneurinilyticus* biomass

**DOI:** 10.3389/fbioe.2024.1452796

**Published:** 2024-08-29

**Authors:** Ksenia Lvova, Xanel Vecino, Benita Pérez-Cid, Ana B. Moldes, José M. Cruz

**Affiliations:** ^1^ Chemical Engineering Department, School of Industrial Engineering-CINTECX, University of Vigo, Campus As Lagoas-Marcosende, Vigo, Spain; ^2^ Department of Analytical and Food Chemistry, Faculty of Chemistry-CINTECX, University of Vigo, Campus As Lagoas-Marcosende, Vigo, Spain

**Keywords:** fermentation, *Bacillus*, antibiotic, ethanol, buffer phosphate

## Abstract

Currently, Gramicidin S (GR-S) is produced enzymatically with the drawback of the presence of trifluoroacetic acid (TFA) or produced by fermentation involving several separation and purification steps. Therefore, this study is focused on the use of green solvents as unique extraction step to produce Gramicidin S from microbial biomass of *Aneurinibacillus aneurinilyticus.* Among the tested solvents, such as ethanol, acidic ethanol or buffer phosphate, the most favorable was acidic ethanol, extracting 96% of Gramicidin S from cells with a purity of 90%. Using acidic ethanol, extraction time within the range of 30–120 min exhibited minimal impact on Gramicidin S yield, whereas the biomass-to-extractant ratio emerged as a critical parameter. Gramicidin S extracts were characterized using Fourier Transform Infrared Spectroscopy (FTIR), Matrix Assisted Laser Desorption/Ionization Time-of-Flight Mass Spectrometry (MALDI-TOF-MS), and Electrospray Ionization Mass Spectrometry (ESI-MS) coupled with Ultra Performance Liquid Chromatography (UPLC) and compared with commercial Gramicidin S.

## Introduction

Gramicidin is a polypeptide antibiotic that can act as a potent therapeutic antimicrobial agent. It is active against gram-positive bacteria, except for *Bacilli*, and against some strains of gram-negative bacteria such as *Escherichia coli* (Wenzel et al., 2018). This antibiotic was discovered 80 years ago (Dubos and Hotchkiss, 1941; Gause and Brazhnikova, 1944), although it has been poorly included in pharmaceutical formulations, limiting its application to ophthalmological or dermal applications because it can induce hemolysis at concentrations lower than those that cause bacterial death. As the outer cells of the epidermis are dead, local application of Gramicidin does not alter the skin surface (Swierstra et al., 2016). Common infections that can be treated with Gramicidin include conjunctivitis, keratitis and blepharitis and it is also used in creams for treating diverse skin infections in combination with other antibiotics like Neomycin and Nystatin. However, recent studies have shown the anti-tumor activity of Gramicidin on human cervical tumor cells (Cabral-Romero et al., 2021) and the cytotoxic effect in human breast cancer cells (Xue et al., 2022). In addition, these antimicrobial peptides are a promising alternative to conventional antibiotics since there is no evidence of resistance toward Gramicidin (Wenzel et al., 2018; Pavithrra and Rajasekaran, 2020; David and Rajasekaran, 2015; Wang et al., 2012). This antibiotic exists in two different chemical forms, linear and cyclic. Linear Gramicidin, also named Gramicidin D, is a mixture of Gramicidin A, B, and C in proportions of approximately 80%, 5%, and 15%, respectively, with molecular weights around 1881–1909 Da depending on the amino acid moiety (Orwa et al., 2001). They are linear peptides with 15 amino acids (David and Rajasekaran, 2015), Gramicidin A (GR-A) being the more abundant antibiotic in the mixture. In contrast, the chemical structure of Gramicidin S (GR-S) is a cyclic decapeptide consisting of ten amino acids with molecular weights between 1,141–1,169 Da, depending on the amino acid moiety or even depending on the union with other molecules like sugars, different ions, or others (Pittenauer et al., 2006; Alenezi et al., 2017; Erve et al., 2009). However, different Gramicidin S homologous can exhibit different bioactivity (Abraham et al., 2014). These antibiotics can be produced naturally as a secondary metabolite by the *Bacillus* strains (Kratzschmar et al., 1989; Kelkar and Chattopadhyay, 2007), like *Bacillus brevis*, which was later reclassified as *Aneurinibacillus migulanus* (Goto et al., 2004). Moreover, López-Prieto et al. (2021) detected the presence of GR-S in a cell-bound biosurfactant extract produced by *Aneurinibacillus aneurinilyticus*, which belongs to the same *Aneurinibacillus* group along with *A. migulanus* (Kamli et al., 2021). However, the authors did not go deeper into the study and no yields or concentration of Gramicidin was provided.

One of the greater drawbacks of the antibiotic production by the Gramicidin producer strains is the downstream process, given that it was observed that Gramicidin is accumulated in cell vacuoles, which serve as energy storage devices (Berditsch et al., 2017). Hence, extraction and purification methods play an important role in obtaining high purity drug extracts. For Gramicidin extraction from *Bacillus* cells, some studies have described the combination of several methods including extraction with organic solvents, acidic precipitation or cell wall disruption summarized in Supplementary Table S1. In any case, the antibiotic efflux from bacterial cells through the extracellular media to the solvent phase is required during the extraction process.

Some authors have proposed the use of complex processes including acetone and ether for Gramicidin extraction followed by evaporation and crystallization steps (Hotchkiss and Dubos, 1941; Nesteruk and Syrov, 2017). Other authors also suggest that Gramicidin extraction with ethanol requires some complex pre-extraction procedures, such as cell culture acidification by HCl (Nesteruk and Syrov, 2017), cell storage at low temperatures (−20°C) followed by incubation of cells in a mixture of NaCl and HCl (Berditsch et al., 2007; Berditsch et al., 2015) or even biomass treatment with acetone (Nesteruk and Syrov, 2017). In addition, a purification process is often needed because Gramicidin is extracted simultaneously with other antibiotics or secondary metabolites from bacterial cells (Bérdy J, 2005).

To make the extraction procedure greener and more sustainable, the selection of a simple process involving environmentally ecofriendly solvents should be preferred (Prat et al., 2014). In this sense, in a previous work, a mixture of ethanol and HCl at high temperatures (70°C) was employed to extract Gramicidin produced by *A. migulanus* (strain Nagano) (Alenezi et al., 2017). Most studies were conducted at temperatures above 40°C; however, a recent research has shown that such high temperatures can lead to the polymerization of Gramicidin S (Pfukwa et al., 2023). In other studies, buffer solution was used for extracting biosurfactants from cells of *Lactobacillus pentosus* obtained from vineyard pruning wastes (Vecino et al., 2015) and from *A. aneurinilyticus* strain present in corn steep liquor (CSL), a residual stream of the corn milling industry (López-Prieto et al., 2021). However, so far there are no studies focused on Gramicidin extraction from microbial biomass of *A. aneurinilyticus* by means of simple processes using sustainable solvents at room temperature. Therefore, it would be interesting to investigate the use of simple and green methods to obtain a high purity Gramicidin antibiotic, especially in the case of GR-S that has a high market price ranging from €67–72/mg (MedChemExpress, 2024; Abmole, 2024).

Considering the above information, this study aims to develop a straightforward extraction method utilizing environmentally friendly solvents to procure a high-purity Gramicidin extract from a wild *A. aneurinilyticus* strain isolated from CSL. A preliminary prescreening consisted of testing of acidic and non-acidic ethanol, along with phosphate buffer saline (PBS), to select the most suitable extractant. Subsequently, the optimal extractant was chosen for determination of the maximum concentration of Gramicidin through consecutive extraction cycles. Optimization of extraction conditions with the preferred extractant involved varying extraction times and ratios of fermented medium containing biomass to extractant. To analyze the extracts, Fourier Transform Infrared Spectroscopy (FTIR), Matrix Assisted Laser Desorption/Ionization Time-of-Flight Mass Spectrometry (MALDI-TOF-MS), and Electrospray Ionization Mass Spectrometry (ESI-MS) coupled with Ultra Performance Liquid Chromatography (UPLC) are employed.

## Experimental section

### Gramicidin production by *A. aneurinilyticus* in a synthetic medium

For Gramicidin production, a 250 mL Erlenmeyer flask containing 100 mL of Tryptic Soy Broth (TSB) was sterilized in autoclave (P Selecta PRESOCLAVE II, Spain) at 121°C for 15 min and the lyophilized strain of *A. aneurinilyticus* (CECT 9939) was inoculated into the synthetic medium. The bacterial inoculum (1 mL) obtained after 48 h of fermentation in TSB at 37°C on a rotary shaker (IKA, Spain), at 150 rpm was used to start the fermentation process in a similar volume of fresh autoclaved TSB. The fermentation process lasted 7 days with the aim of achieving the stationary phase under the same conditions mentioned above. Moreover, the colonies of *A. aneurinilyticus* were isolated from fermented TSB medium on a plate with Tryptic Soy Agar (TSA) to check their phenotype during the stationary phase.

### Quantification of microbial biomass

As Gramicidin is contained in vacuole cells biomass was quantified gravimetrically by using a conventional oven to dry the microbial biomass produced during fermentation of TSB medium. Thus, the biomass of the fermented medium (1 mL) was centrifuged and twice washed with Milli-Q water. The amount of the biomass obtained was placed into pre-dried and pre-weighed glass tubes and left at 105°C in a conventional oven for 48 h (Bustos et al., 2018). After drying, the final weight of the biomass formed was determined by weight difference of the sample tubes using an analytical balance (Denver Instrument) and expressed as grams of dry biomass formed per liter of the fermented medium. In some assays, the biomass was evaluated for a period of 7 days fermentation to detect the stationary phase. It is important to indicate that the experiments were conducted in triplicate and the results were expressed as the mean value and standard deviation of the three independent cultures.

### Gramicidin extraction

**FIGURE 1:**
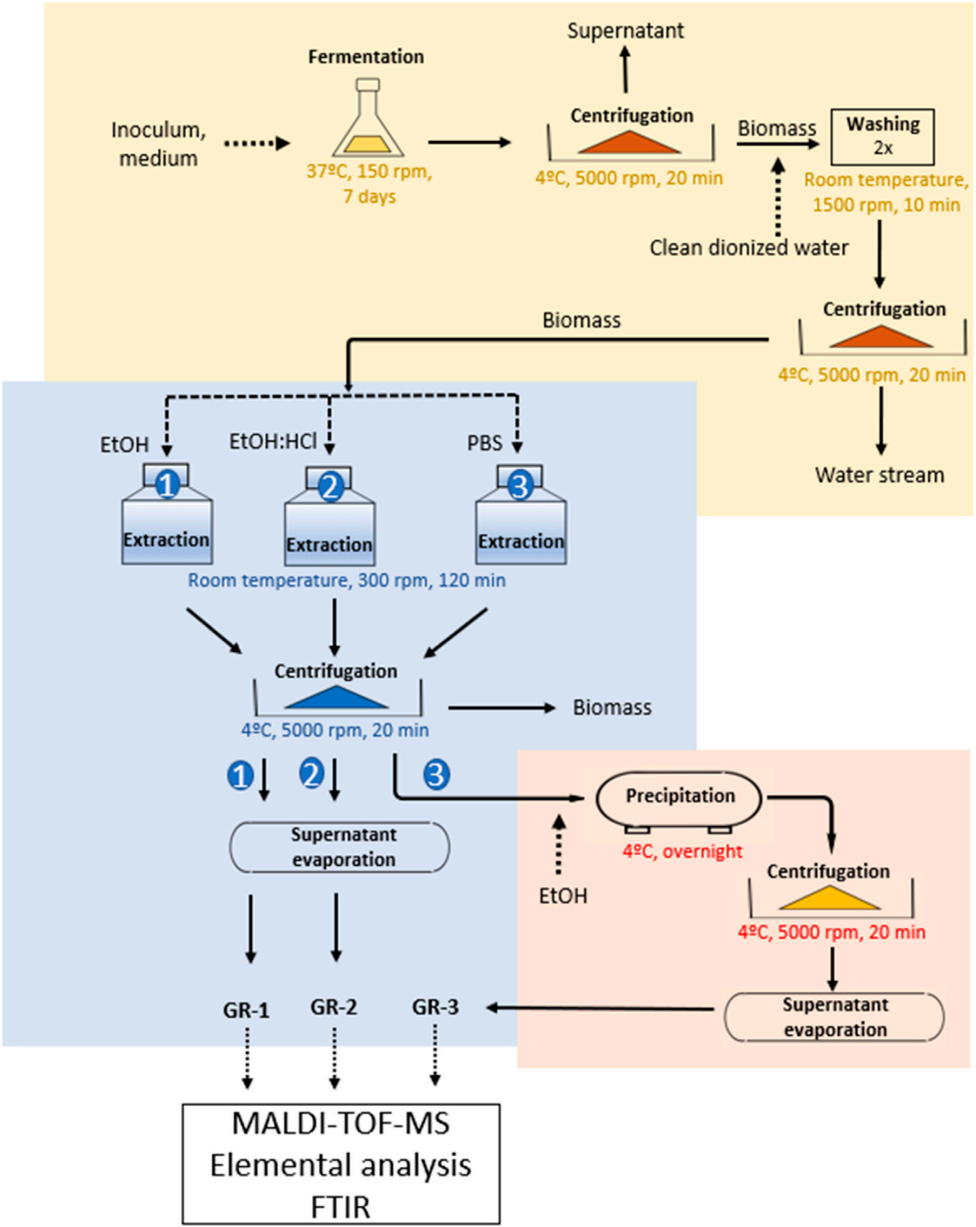
Scheme of Gramicidin extraction from the microbial biomass.

Gramicidin was extracted by solid-liquid extraction of the microbial biomass after fermentation of TBS medium. Figure 1 shows the scheme of Gramicidin extraction from biomass followed by a purification proposal of the extracts.

Thus, the fermented TSB medium (300 mL) was collected and centrifuged (5,000 rpm, 20 min, 4°C) and the resulted biomass was then washed twice with Milli-Q water and subjected to solid-liquid extraction of Gramicidin with 100 mL of different extractant reagents such as phosphate buffer solution (PBS) composed by 10 mM KH_2_PO_4_/K_2_HPO_4_ and 150 mM NaCl, ethanol (Berditsch et al., 2015), or a mixture of ethanol and 0.2 N hydrochloric acid (with a ratio 9:1 (v/v)) (Fang et al., 1997). The extraction process occurred for 2 hours of constant agitation at room temperature in a shaker at 300 rpm. When the extraction process was over, the PBS and ethanol solutions containing Gramicidin were subjected to the same centrifugation step described previously and the final Gramicidin extracts of ethanol (GR-1) and ethanol:HCl (GR-2) were analyzed by elemental analysis, FTIR, and MALDI-TOF-MS as indicated in Figure 1.

Moreover, aiming to remove salts contents, the PBS-Gramicidin extract (GR-3) was subjected to a further purification process by adding ethanol to the PBS- Gramicidin extract in a ratio 7:3 (v/v) overnight, at 4°C. This process was carried out to facilitate the deposition of salts and other impurities as proposed by Maione et al. (2016) to isolate GR-S or GR-A from a PBS matrix. After precipitation of salts, the sample was centrifuged and the supernatant (GR-3) was submitted to elemental analysis, FTIR, and MALDI-TOF-MS analysis following the scheme in Figure 1.

The mass of Gramicidin extracts obtained with ethanol, ethanol:HCl, or PBS was quantified by means of a gravimetric method consisting of drying a volume (1 mL) of the extract in a conventional oven following the procedure described previously (Bustos et al., 2018).

### Elemental analysis of Gramicidin extracts during preliminary screening

Elemental analysis of carbon, hydrogen, and nitrogen in the different Gramicidin extracts was done by means of an elemental analyzer (Fisons Carlo Erba EA-1108 CHNS-0, LabX, Midland, ON, Canada). The protein content in the samples was calculated by multiplying the nitrogen content with the nitrogen-to-protein conversion factor (6.25) (Mariotti et al., 2008).

### Characterization of Gramicidin extracts by FTIR during preliminary screening

Different Gramicidin extracts were chemically characterized by Total Reflectance-Fourier Transform Infrared Spectroscopy (ATR-FTIR) using a Nicolet 6700 FTIR spectrometer (Thermo Scientific) which is coupled with a Smart Orbit ATR featuring a diamond crystal mounted on a tungsten carbide support. The crystal has a refractive index of 2.40 at 1,000 cm^−1^ and an incidence angle of 45° (single reflection). The equipment includes a KBr beam splitter and a deuterated triglycine sulfate (DTGS) detector. Samples are measured with 34 scans, over a range of 400–4,000 cm^−1^, with a resolution of 4 cm⁻^1^. Moreover, the resulting FTIR spectra of Gramicidin extracts were compared with commercial GR-A and commercial GR-S. The similarity indexes (P) of samples were obtained by comparison with commercial Gramicidin based on the Pearson correlation factor (*r*_*P*_) and the Spearman correlation factor of (*r*_*S*_) proposed by Henschel et al. (2020). Therefore, correlation factors for samples were obtained taking into consideration the spectral measurements made in the transmittance mode in the range of 400–4,000 cm^-1^. The factor *r*_*P*_ was calculated directly with the Excel statistical tool, whereas the factor *r*_*S*_ was obtained using Equation 1.

$$rs=1-\frac{6\sum_{i}d_{i}^{2}}{n\left(n^{2}-1\right)}$$ (1)

where *d*_*i*_ is the difference between the ranks of the transmittance values compared in their respective data set and *n* the number of elements in each vector.

Similarity indexes P_p_ and P_s_ were calculated based on *r*_*P*_ and *r*_*S*_ following Equation 2:

$$P\;\left(\%\right)=r^{2}\;100$$ (2)

where P is the percentage of similarity index and *r* is the correlation coefficient of Pearson (*r*_*P*_) or Spearman (*r*_*S*_), respectively. Hence, the similarity index depending on the correlation coefficient used, Pearson or Spearman´s, was named P_p_ or P_s,_ respectively.

### Analysis of Gramicidin extracts by MALDI-TOF-MS during preliminary screening

Gramicidin extracts were analyzed by Matrix Assisted Laser Desorption/Ionization Time-of-Flight Mass Spectrometry (MALDI-TOF-MS). The analysis was carried out by means of a dried droplet sample preparation method consisting of applying 1 µL of α-cyano-4-hydroxycinnamic acid (CHCA) matrix solution (3 mg/mL in 50% ethanol (v/v)) directly on a MTP AnchorChip™ 800/384 TF MALDI target (Bruker Daltonik, Bremen Germany) and, before drying the matrix solution, 1 µL of sample in ethanol was added and allowed to dry at room temperature. External mass calibration was performed with a calibration standard (Bruker Daltonik, Bremen Germany) for the range *m/z* 700–3,000 (9 mass calibrant points). Thus, 0.5 mL of the stock solution and CHCA matrix, previously mixed in an Eppendorf tube 1:2 (v/v), was applied directly on the target and allowed to dry at room temperature. Mass spectra were recorded using an Autoflex III smartbeam MALDI-TOF mass spectrometer Bruker Daltonik (Bremen, Germany), operating in reflector positive ion mode as described in a previous work (Rincón-Fontán et al., 2017).

### Optimization and validation of selected extraction conditions

In order to optimize extraction conditions of the selected method, Gramicidin extracts were obtained by using acidic ethanol, as described previously in section “Gramicidin extraction and purification”, but varying the extraction time (30, 60, 120 min) and the ratio fermented medium containing biomass to extractant (3:1 and 6:1) v/v. Additionally, GR-2 extract was obtained at 40°C during 120 min with 3:1 v/v and the samples were analyzed by MALDI-TOF-MS following the procedure described previously in section “Analysis of Gramicidin extracts by MALDI-TOF-MS”.

To validate the results obtained during the prescreening, Gramicidin in the extracts was quantified by Ultra Performance Liquid Chromatography-tandem Mass Spectrometry (UPLC-MS/MS) using Electrospray Ionization Mass Spectrometry (ESI-MS) as ions source (Mass Bruker FTMS SolariX XR) coupled with Ultra Performance Liquid Chromatography (UPLC), elute from Bruker equipped with degasser, binary pump, column oven and automatic injector. A column Zorbax Eclipse Plus Phenyl-Hexyl, rapid resolution HD 2.1 × 100 mm (1.8 µm) column operating at 30°C with a mobile phase flow rate of 500 μL/min and using the following gradient program (% B; time) (44% B; 0–6 min) (98% B; 6.1–8 min) (44% B; 8.1–10 min). Solvent A was Milli-Q water with 0.1% formic acid and solvent B was acetonitrile with 0.1% formic acid. For standards and samples, a constant injection volume of 5 µL was used. Mass spectrometer was operated in positive electrospray ionization (ESI) mode in a mass range between 100 and 2,000 Da. The capillary was set to 4500 V and the end plate offset to 500 V. Nitrogen was employed as nebulizing gas at a pressure of 8.0 bar as well as drying gas with a flow rate of 8.0 L/min at 220°C.

The purity of GR-S in GR-2 extracts was assessed by comparing the GR-S content quantified by UPLC-MS/MS in each extract with its corresponding dry weight.

### Biological assay of Gramicidin extract obtained under the selected extractive process

To assess the antimicrobial properties of the Gramicidin extract GR-2, a disc diffusion bioassay was utilized. Tryptic soy agar (TSA) plates were prepared and inoculated with *Bacillus licheniformis,* belonging to the phylogenetic group of *Bacillus subtilis* and identify as *B. licheniformis* CICC 23972, serving as the antagonist strain. This strain was previously isolated from the commercial corn steep liquor (Feed Stimulants, the Netherlands) and identified by MALDI-TOF-MS using a Mass Spectrometer Microflex L20 (Bruker Daltonics) equipped with a laser N2.

The plates were placed in an incubator set at 37°C for 48 h. Small disks of filter paper, measuring 6 mm in diameter, were soaked in neutralized solutions of GR-2 at concentrations of 1 g/L and 3 g/L. The positive control was prepared using commercial GR-S at a concentration of 1 g/L, dissolved in the same solvent as GR-2. For the negative control, neutralized ethanol was used, mirroring the solvent matrix of both GR-2 and commercial GR-S. Each sample disk contained 10 µL of the respective solution.

### Statistical analysis

Statistical analysis of data was performed by means of the SPSS 24.0 Statistics software (IBM Corporation, Armonk, NY, USA). One-way analysis of variance (ANOVA) using the Tukey-b *post hoc* test (*p* < 0.05) was employed to evaluate significant differences among the results compared along the current work.

## Results and discussion

### Production and evaluation of *A. aneurinilyticus* microbial biomass

Given that Gramicidin is accumulated in cell vacuoles (Berditsch et al., 2017), the microbial biomass produced by *A. aneurinilyticus* is the main source of this antibiotic. For this reason, the amount of microbial biomass produced during a period of incubation of 7 days at 37°C in the TSB fermentation medium was evaluated (Supplementary Figure S1). The dry weight of biomass was expressed in g/L. Mean values and their standard deviations were calculated from three independent cultures grown in TSB medium.

The graph and accompanying images of flasks illustrating 2, 3, 5, and 7-day fermented TSB show a notable increase in microbial biomass growth during the initial 4 days of incubation as observed in Supplementary Figure S1A. This growth pointed at 3.3 g/L and remained relatively stable through day 7.

Therefore, a period of incubation higher than 5 days was selected in this work to ensure maximum microbial growth, as spore forming *Bacillus* strain usually produce Gramicidin as a secondary metabolite during their stationary phase. These results are in good agreement with those reported in a previous work, where the incubation conditions used to produce GR-S from *Aneurinibacillus migulanus* strain Nagano were 37°C during a period of 96 h in a synthetic medium (Alenezi et al., 2017). In a previous study (Schuster and Schmitt, 2018) focused on the antifungal activity of *A. migulanus*, the bacterial culture was also produced after inoculation in TSB medium overnight, followed by 5 days of incubation at 37°C. Moreover, the results provided by Berditsch et al. (2015) revealed that Gramicidin production starts when the stationary phase is reached, obtaining between 2.2 and 4.3 g/L of dry weight of biomass from a complex nutritional medium.

Further, the colonies of *A. aneurinilyticus* were isolated from fermented TSB medium during the stationary phase of growth and the pictures of the bacteria were taken (Supplementary Figure S1B). It can be noticed in the pictures (Supplementary Figure S1B), the morphology of these isolated colonies is characterized by a beige-orange color with a pronounced rugose center that is like the description given by Berditsch et al. (2007) for the original Gramicidin producing phenotype of *A. migulanus*. Moreover, the diameter of the representative colonies measured between 4 and 7 mm that it is in consonance with the data provided by Berditsch et al. (2007) for *A. migulanus*. In taxonomic hierarchy, both species *Aneurinibacillus aneurinilyticus*, also known as *Aneurinibacillus aneurinolyticus* or *Bacillus aneurinolyticus* and *Aneurinibacillus migulanus* belong to the class of Bacilli and the genus of Aneurinibacillus (Shida et al., 1996).

### Preliminary evaluation of different extractive methods for obtaining greener Gramicidin extracts from *A. aneurinilyticus*

The selection of the correct extractant and the use of mild extraction conditions are important factors to obtain greener bioactive compounds including antibiotics. In this work, simple extraction procedures were employed where the methods consisted in the treatment of the water-washed microbial biomass with ethanol, ethanol:HCl or PBS solution in one-step extraction at room temperature (Figure 1), as described in the section regarding the extraction and purification of Gramicidin. Mild conditions prevent the oligomerization of Gramicidin S, as reported in a previous study (Pfukwa et al., 2023) and reduce the energy consumption as well. The yield of each Gramicidin extract (GR-1, GR-2 and GR-3) was shown in Table 1.

**TABLE 1:** The yield of Gramicidin extracts relative to the fermented medium and relative to the biomass, obtained with ethanol, ethanol:HCl and PBS using a synthetic medium TSB.

Gramicidin extract	Gramicidin S (g/L)^a^	Gramicidin S (g/g)^b^
GR-1	0.617 ± 0.13	0.185 ± 0.04
GR-2	1.167 ± 0.28	0.349 ± 0.08
GR-3	0.072 ± 0.05	0.022 ± 0.02

^a^ Relative to the fermented medium.

^b^ Relative to the biomass.

These results are expressed as dry weight of the extract per liter of fermented liquid and per gram of biomass. Moreover, the data represent average values of two determinations and their standard deviation. The yield of Gramicidin extracts respect to the fermentation medium were 0.671 g/L, 1.167 g/L, and 0.072 g/L of GR-1, GR-2 and GR-3, respectively. The amount of sample GR-3, free of salt, obtained was determined by deducting the salts present in PBS (11.86 g/L) from the dry weight of the crude GR-3 (12.08 g/L), as the purification step with ethanol was not able to remove all the salts. The yield of crude GR-3 was notably low, suggesting a limited extraction of bio-compounds from the microbial biomass.

On the other hand, the yield of Gramicidin extracts respect to *A. aneurinilyticus* biomass were 0.185 g/g, 0.349 g/g, 0.022 g/g of GR-1, GR-2, and GR-3, respectively. Among the extraction processes assayed in this work, acidic ethanol produced almost twice as much Gramicidin extract compared to non-acidic ethanol. Taking into consideration these results, a mixture of ethanol and HCl was employed to produce Gramicidin extracts from the biomass that was collected after 3 days and 7 days of fermentation in TSB medium to evaluate the effect of fermentation time on the production and extraction of Gramicidin regarding the biomass. The yield of the extracts after 3 days and 7 days were 0.800 g/L and 1.300 g/L, respectively. The amounts of these extracts were 0.301 g/g and 0.389 g/g for three and 7 days of fermentation in TSB, respectively. It was observed that during the exponential phase *A. aneurinilyticus* also produces Gramicidin, although a higher yield was observed, respect to the biomass concentration, during the stationary phase. It is likely that the decrease of nutrients in the stationary phase induced a higher antibiotic production. This aligns with findings from other researchers. For example, Berditsch et al. (2015) observed that *Aneurinibacillus migulanus* began producing Gramicidin S after entering the stationary phase when the maximum yields were achieved, following the highest consumption of phenylalanine used as an inducer for Gramicidin S production.

Furthermore, the different Gramicidin extracts (GR-1, GR-2, and GR-3) were subjected to elemental, FTIR, and MALDI-TOF-MS analysis. Table 2 shows the results of elemental analysis, expressed as mean value and standard deviation of three determinations, observing that both ethanol (GR-1) and ethanol:HCl extracts (GR-2) have percentages of nitrogen (9.11%–10.61%), carbon (52.15%–55.32%), and hydrogen (7.55%–7.66%) consistent with the elemental analysis of commercial GR-A (13.80% N, 58.04% C and 6.36% H) or GR-S (11.44% N, 52.82% C, and 7.07% H) considered as reference values.

**TABLE 2:** Elemental analysis of Gramicidin extracts in comparison with the commercial GR-A and GR-S.

Gramicidin extract	N (%)	C (%)	H (%)	C/N
GR-1	9.11 ± 0.16^a^	55.32 ± 1.03^a^	7.66 ± 0.61^a^	6.07 ± 0.10^a^
GR-2	10.61 ± 0.10^b^	52.15 ± 0.57^b^	7.55 ± 0.34^a^	4.92 ± 0.08^b^
GR-3	0.055 ± 0.01^c^	0.30 ± 0.01^c^	0.28 ± 0.01^b^	5.45 ± 0.45^c^
GR-A	13.80 ± 0.16^d^	58.04 ± 2.21^d^	6.36 ± 0.57^c^	4.20 ± 0.11^d^
GR-S	11.44 ± 0.15^e^	52.82 ± 0.29^b^	7.07 ± 0.34^a,c^	4.62 ± 0.04^b^

Different Latin letters within the same column indicate significant differences among Gramicidin extracts according to Tukey-b test for ANOVA, analysis (*p < 0.05*).

Moreover, these data are in consonance with the values reported by Hotchkiss and Dubos (Hotchkiss and Dubos, 1941), for the linear Gramicidin extract obtained from *B. brevis* after three recrystallization cycles with acetone, that was composed of 59.6% C, 6.66% H, 14.31% N.

In the context of the current study, the PBS extract (GR-3) obtained from *A. aneurinilyticus* exhibited nitrogen, carbon, and hydrogen percentages consistently below 1% post-ethanol purification, notably lower than those observed in the ethanol extracts GR-1 and GR-2 (refer to Table 2). These reduced values of C and N can be attributed to the significant presence of salts, persisting even after purification, originating from the phosphate and sodium chloride salts present in PBS. Additionally, the low extraction yield can be explained from the limited solubility of Gramicidin in aqueous solutions (Solovskij and Panarin, 1999), impeding its solubilization in PBS. The statistical treatment of the data by ANOVA analysis indicates a great similarity between the results of commercial GR-S and the ethanol:HCl extract (GR-2) with no significant differences (Tukey-b test; *p* < 0.05) for carbon, hydrogen, and the C/N ratio. Only significant differences were found for the content of nitrogen; although the values were very similar with differences of only 0.83% between both commercial GR-S and GR-2 (10.61% N for GR-2 extract and 11.44% N for commercial GR-S). In the case of the ethanol extract (GR-1), only the percentage of hydrogen (7.66%) was statistically equivalent at the values obtained for commercial GR-S (7.07%). From the statistical analysis of the results coming from the elemental analysis, it can be speculated that GR-2 is composed mainly of GR-S, whereas GR-1 could contain a higher amount of other bioactive compounds although given the relative proximity in the percentages of C and N and the good agreement in the percentage of H, following the ANOVA analysis, GR-S should also be present in GR-1 to a great extent. However, according to the elemental analysis the presence of GR-A could not be discharged at this point in GR-1 and GR-2.

**FIGURE 2:**
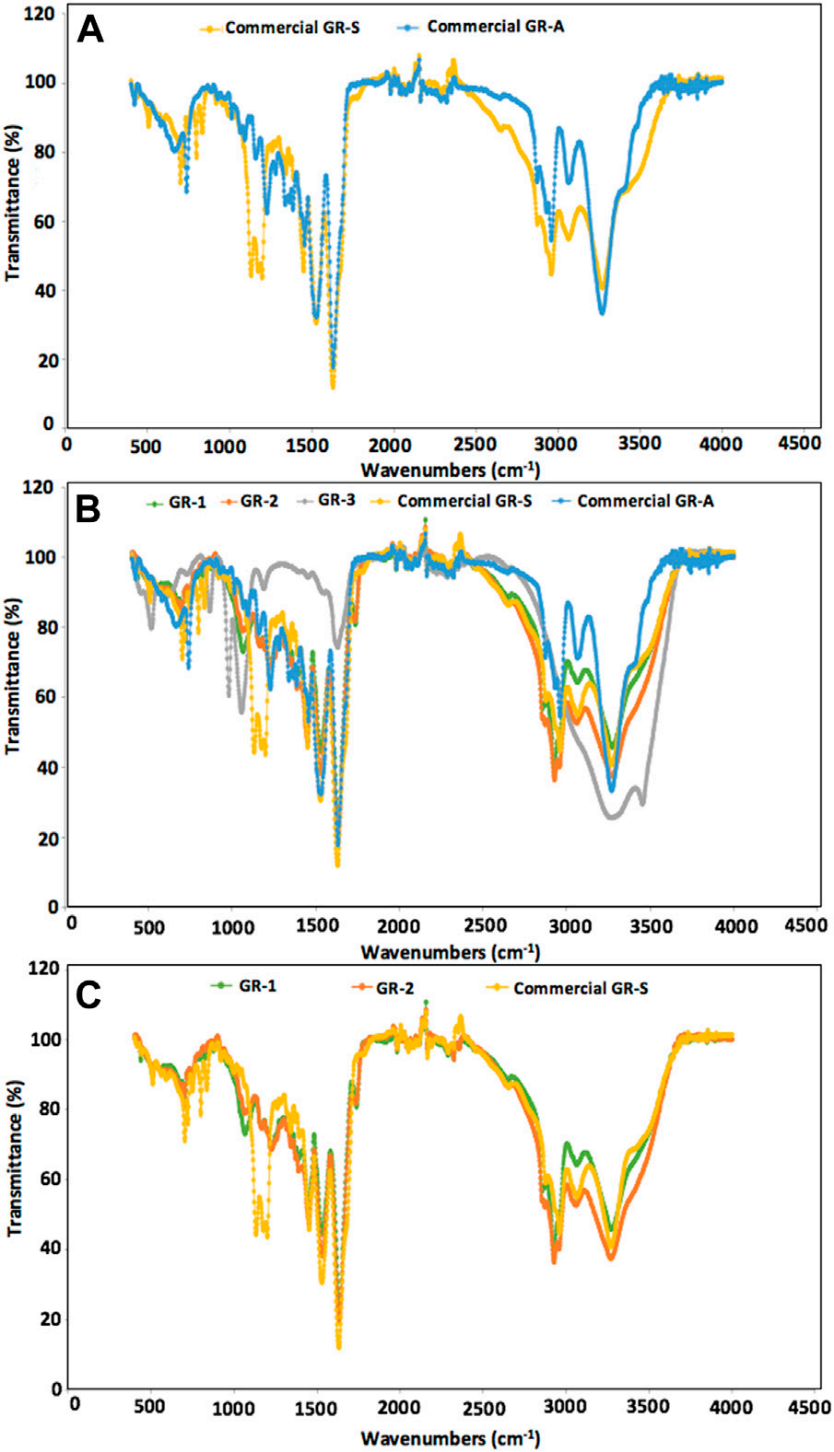
Comparison of FTIR spectra between 400–4,000 cm^−1^ of: **(A)** Commercial GR-A and GR-S; **(B)** Commercial GR-S and GR-A with GR-1, GR-2, and GR-3; **(C)** Commercial GR-S with GR-1 and GR-2.

Aiming to identify the functional groups and the chemical bonds in the different Gramicidin extracts, the FTIR spectra of Gramicidin extracts were obtained between 400–4,000 cm^−1^ and compared with both commercial GR-A and GR-S spectra. Hence, Figure 2 presents the FTIR spectra of the commercial GR-A and GR-S (Figure 2A) and the GR extracts under evaluation (GR-1, GR-2 and GR-3) (Figure 2B) whereas in order to obtain a better visualization, the FTIR spectra of GR-S and the extracts GR-1 and GR-2 are compared in Figure 2C between 400–4,000 cm^−1^, the highest similarity index being observed between the commercial Gramicidin products and ethanol-Gramicidin extracts.

In general, when GR-1 and GR-2 were compared with GR-S, there was seen to be a good similarity between the two ethanol-Gramicidin extracts and GR-S, with correlation factors r_p_ and r_s_ higher than 0.928 and 0.951, respectively, corresponding with similarity indexes higher than 86.1% and 90.5%, respectively (Figure 2; Table 3).

**TABLE 3:** Correlation coefficients of Pearson (*r*_*p*_) and Spearman (*r*_*S*_) and similarity index (P_p_) and (P_s_) of Gramicidin crude extracts obtained from *Aneurinibacillus aneurinilyticus* in comparison with commercial GR-A and GR-S.

Comparison with commercial GR-S (400–4,000 cm^−1^)				
	r_p_	% P_p_	r_S_	% P_S_
GR-1	0.930	86.55	0.952	90.71
GR-2	0.928	86.07	0.951	90.50
GR-3	0.619	38.30	0.589	34.72
GR-A	0.891	79.38	0.910	82.87

Comparison with commercial GR-A (400–4,000 cm^−1^)				
	r_p_	% P_p_	r_S_	% P_S_
GR-1	0.909	82.66	0.918	84.29
GR-2	0.892	79.48	0.905	81.95
GR-3	0.536	28.71	0.664	44.04

Comparison with commercial GR-S (1,250–4,000 cm^−1^)				
	r_p_	% P_p_	r_S_	% P_S_
GR-1	0.971	94.11	0.966	93.33
GR-2	0.971	94.21	0.967	93.57
GR-A	0.930	86.40	0.922	85.08

Moreover, a perfect match can be observed between GR-1 and GR-2 (Figure 2C), demonstrating that the use of HCl facilitated the release of Gramicidin S from the cells without affecting the liberation of other compounds what it is in consonance with the results of the elemental analysis included in Table 2. The similarity between GR-1 and GR-2 with GR-S was even higher than the similarity shown between commercial GR-S and GR-A (Figure 2; Supplementary Figure S2; Table 3). Furthermore, when GR-1 and GR-2 were compared with GR-A, high similarity indexes were also detected, although lower than that observed with GR-S (Table 3). The highest similarity index was obtained between GR-1 and GR-A with a r_p_ and r_s_ values of 0.909 and 0.918 corresponding with similarity indexes P_p_ and P_s_ of 82.66% and 84.29%, respectively. This is because GR-S and GR-A are composed of similar functional groups and similar amino acids, thus it is normal to obtain high similarity indexes between GR-S and GR-A.

The results regarding elemental analysis of commercial GR-S are consistent with the similarity between FTIR spectra of commercial GR-A and GR-S where the similarity index is in the range between 79.38% (P_p_) and 82.87% (P_s_), depending on the correlation factor used (Table 3). The high similarity index is unsurprising, as the functional groups of amino acids present in both antibiotics are expected to be similar.

On the other hand, in the FTIR of commercial GR-S a pronounced band is observed between 1,100 and 1,200 cm^−1^ produced by the functional groups of the Trifluoroacetate (TFA) contained in commercial GR-S (Figure 2A). If this band is subtracted, an increase in similarity index between GR-S, GR-1 and GR-2 can be observed in Supplementary Figure S2. It is known that synthetic peptide or protein samples are mostly unpurified with TFA salt (Valenti et al., 2011). TFA salt is used during the enzymatic production of GR-S. This method has the inconvenience of its elevated cost and the presence of salt in the final product which could prevent its use in the pharmaceutical industry. Despite that, commercial GR-S used in this work as control has an elevated cost ranging from €67–72/mg [https://www.medchemexpress.com/gramicidin-s.html; https://www.abmole.com/products/gramicidin-s.html (accessed 2024–07–18)].

Hence, if the comparison between commercial GR-S and GR-2 is carried out in the zone at 1,250–4,000 cm^−1^, P_P_ and P_S_ can increase up to 94.21% and 93.57%, respectively. Also, the similarity between GR-1 and GR-S increased in this range (Supplementary Figure S2; Table 3). However, in terms of productivity and yield, GR-2 is recommended rather than GR-1 as almost double the amount of Gramicidin is extracted with acidic ethanol in comparison to nonacidic ethanol (Table 1). Moreover, when commercial GR-S and GR-A are compared in the zone at 1,250–4,000 cm^−1^, P_P_ and P_S_ increased up to 86.40% and 85.08% respectively, although these values were lower than those achieved with GR-1 or GR-2.

Finally, the FTIR spectrum of PBS-extract (GR-3) presents a considerably lower similarity index regarding commercial GR-A or GR-S (lower than 50%) (Figure 2B; Table 3) indicating that this extract has a lower purity than ethanolic extracts GR-1 and GR-2, possibly due the presence of salts from PBS and also because the presence of more hydrophilic biocompounds released from the cell membrane that it is consistent with the results of elemental analysis (Table 2).

In the literature, there is little data relating to the purity of the Gramicidin extracts produced biotechnologically. For example, Nesteruk and Syrov (Nesteruk and Syrov, 2017) have obtained a Gramicidin extract using a mutant strain of *A. migulanus* with a related impurity content of 15.3% that is consistent with the data obtained in this work for GR-1 and GR-2.

However, FTIR spectra could not be specific enough to discriminate between the molecular structure of GR-A or GR-S due to the great coincidences in the functional groups and in chemical bonds of their linear or cyclic molecules, respectively. In fact, the FTIR spectra of GR-A and GR-S as well as the FTIR of Gramicidin extracts showed peaks between 3,400–3,200 cm^−1^ due to the presence of the amine (NH) and hydroxyl groups (OH). Moreover, the peaks between 3,000 and 2,800 cm^−1^ indicate the presence of aliphatic chains with abundance of CH, CH_2_ and CH_3_ bonds (Vecino et al., 2015). The peaks around 1,650 cm^−1^ correspond to the presence of CO bonds and the band about 1,535 cm^−1^ is associated with the presence of CN bonds (Freitas de Oliveira et al., 2013).

### Characterization of Gramicidin extracts by MALDI-TOF-MS

MALDI-TOF-MS analyses were applied to discriminate between the presence of GR-A or GR-S in the extracts obtained from *A. aneurinilyticus* growth in TSB medium. MALDI can be considered a highly selective method for the identification of biomolecules, including peptides, proteins, antibiotics, etc (Torres-Sangiao et al., 2021) being a time-effective alternative methodology to conventional procedures. In this way, previous studies have employed other less sensitive and selective analytical methods like spectrophotometry (660 nm) (Bruckner et al., 1995) or High-Pressure Liquid Chromatography (HPLC) with UV detection for quantification of GR-A (Adams et al., 1997) and even HPLC with fluorescence detection for identification of GR-S (Bruckner et al., 1995).

**FIGURE 3:**
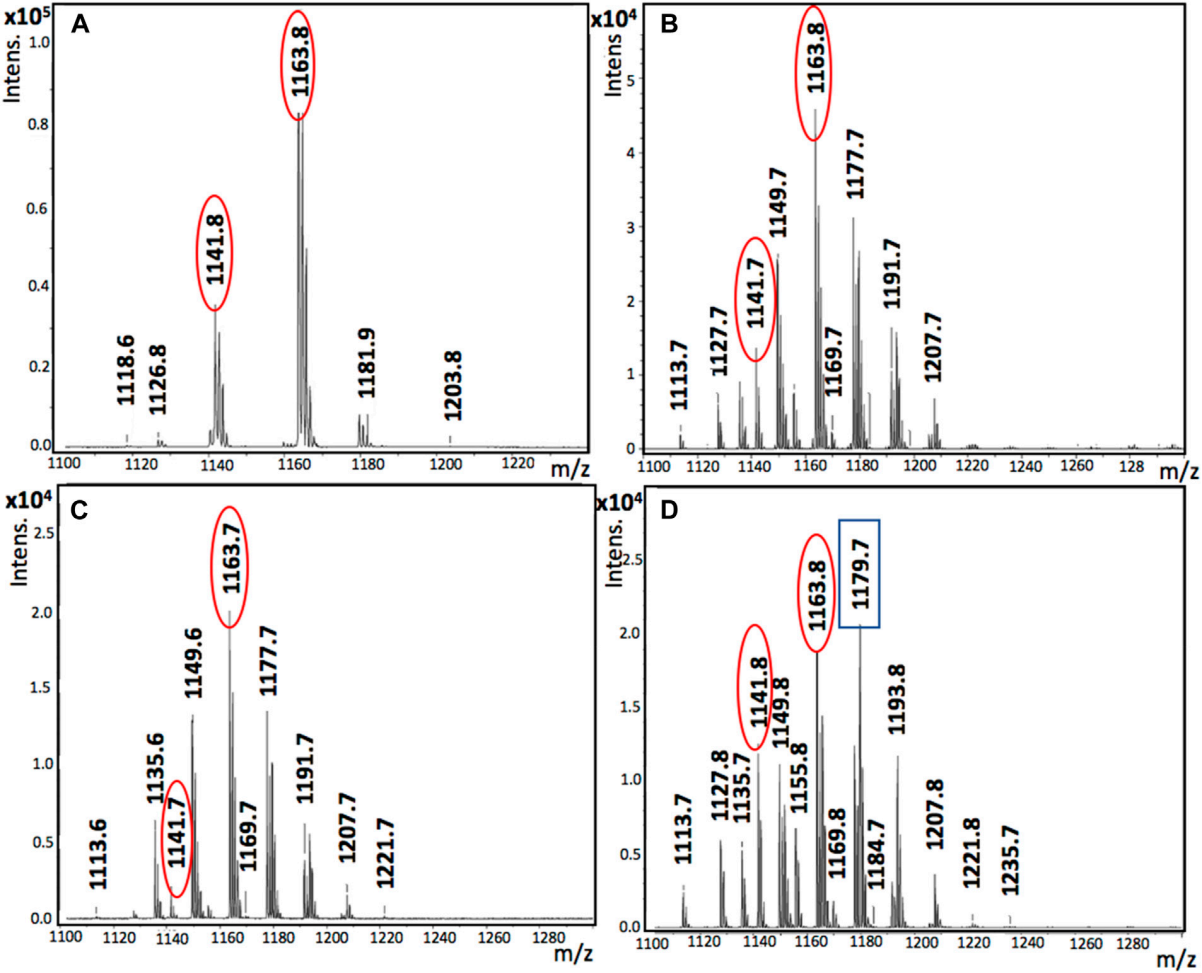
MALDI spectra of commercial GR-S and Gramicidin extracts obtained after extraction process: **(A)** Commercial GR-S; **(B)** GR-1; **(C)** GR-2; **(D)** GR-3.

The MALDI spectra of all Gramicidin extracts analyzed in this study, along with the spectrum of the stock solution of commercial GR-S (10 μg/μL), are presented in Figure 3 for comparison.

The findings indicated the presence of GR-S in all three analyzed Gramicidin extracts (GR-1, GR-2, and GR-3), as evidenced by the detection of mass peaks corresponding to its protonated molecular ion [M^+^H]^+^ at 1,141.6–1,141.8 m/z. This aligns with the mass spectrum of commercial GR-S at 1,141.8 m/z (Figure 3A). The same molecular ion of GR-S was also found in previous works focused on the identification of this antibiotic produced by *A. migulanus* (Pittenauer et al., 2006; Berditsch et al., 2015) or even in the commercial product (Pittenauer et al., 2006). It is important to highlight that the mass peaks of the molecular ion of GR-S associated to sodium [M^+^Na]^+^ at 1,163.7–1,163.8 m*/z* were detected in all the Gramicidin extracts analyzed as well as in the commercial standard (Figure 3). In addition, the mass peak corresponding to the molecular ion of GR-S associated to potassium [M^+^K]^+^ at 1,179.7 m*/z* was only detected in the PBS extract (Figure 3D), probably due to the presence of potassium salts (K_2_HPO_4_ and KH_2_PO_4_) in the PBS solution. These results are in good agreement with those found in a previous work (Pittenauer et al., 2006) based on the identification of commercial GR-S by MALDI-TOF-MS analysis, which also found the mass peaks corresponding to Gramicidin associated to sodium [M + Na]^+^ and potassium [M + K]^+^ ions at values of 1,163 and 1,179.7 m*/z*, respectively. The MALDI spectra results are in line with those obtained from elemental analysis (refer to Table 2), which underwent ANOVA analysis (Tukey-b test *p* < 0.05). However, in the case of the PBS extract (GR-3), although GR-S was detected in the MALDI spectra (Figure 3D), its presence in GR-3 was deemed negligible based on the results from elemental analysis and FTIR spectra. It's worth noting that GR-A was not detected in the range of 1850–1950 m/z in any of the MALDI spectra corresponding to the analyzed Gramicidin extracts.

Based on the results, regarding analysis elemental, FTIR and MALDI spectra, obtained for GR-2 in comparison with GR-1 and GR-3, acidic ethanol was chosen as an extraction method. Using acidic ethanol, it was obtained the highest crude extract yield and the greatest similarity index when compared with commercial Gramicidin S using FTIR analysis. Therefore, in the subsequent part of the study, various operational variables, including extraction time, biomass:extractant ratio, and number of extraction cycles, were evaluated using UPLC-MS/MS to quantify Gramicidin.

### Optimization and validation of extraction conditions based on acidic ethanol

**FIGURE 4:**
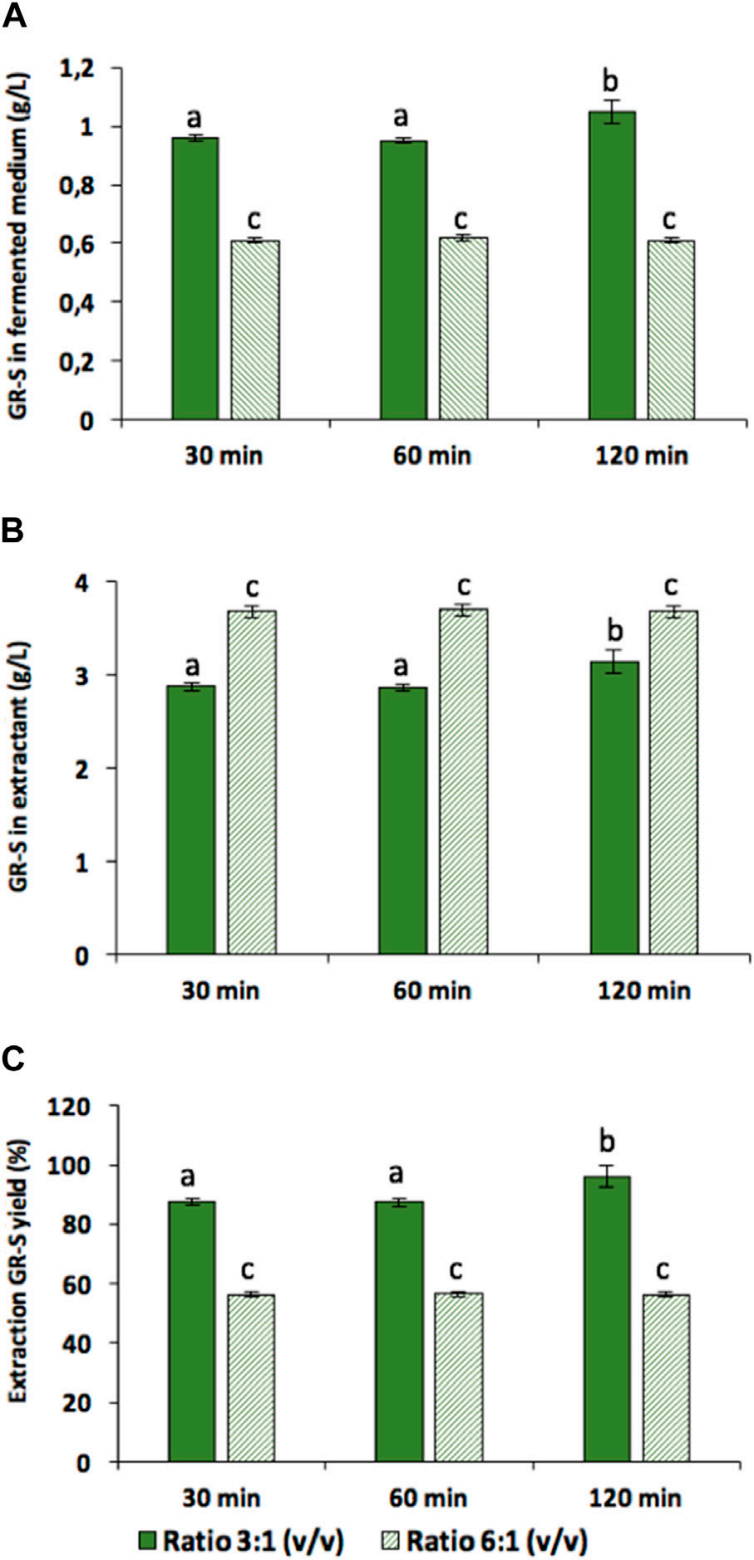
Content of GR-S complex (mg/L) in fermented medium **(A)**; in the extractant **(B)** and extraction percentage yield **(C)**. Different Latin letters within the same graphic indicate significant differences among the results according to the Tukey-b test (*p* < 0.05) for one-way ANOVA analysis.

To optimize GR-2 production, various extraction processes were conducted varying both extraction time (30, 60, and 120 min) and ratio of fermented medium to acidic ethanol (3:1 and 6:1, v/v). These trials include the optimal conditions, such as the extraction ratio 3:1 v/v and 120 min of extraction time, that were determined in prior experiments for Gramicidin extracts in ethanol:HCl. Moreover, maintaining room temperature during extraction process prevents alterations in the conformational structure of GR-S, which can occur at temperature exceeding 40°C (Pfukwa et al., 2023). As depicted in Supplementary Figure S3, the relative abundance of GR-S molecule complexes decreased in the Gramicidin extracts obtained at temperature up to 40°C in comparison with the extracts obtained at room temperature that aligns with findings reported in other study (Pfukwa et al., 2023). Figure 4 illustrates the quantity of GR-S in both the fermentation medium (Figure 4A) and the extractant (Figure 4B). Notably, extraction time did not significantly impact GR-2 yield.

However, the ratio of fermented medium to extractant (v/v) emerged as a critical factor. A substantial decrease in GR-S relative to the fermentation medium was observed when the extraction ratio was set at 6:1 v/v. In these experiments, GR-S analysis was conducted by UPLC- MS/MS. Notably, the doubly charged ion [M+2H]^2+^ of GR-S (m/z of 571.36) exhibited the highest response and was chosen as the precursor ion for quantitative purposes (Supplementary Figure S4 in the Supplementary Material). Calibration curves were established using stock solutions of commercial GR-S ranging from 1 to 10 μg/mL, revealing a linear correlation (y = 8,731,72x - 836,174; r^2^ = 0.9904) between the analytical signal (y = peak area) and the concentration of GR-S (x = µg/mL).

**FIGURE 5:**
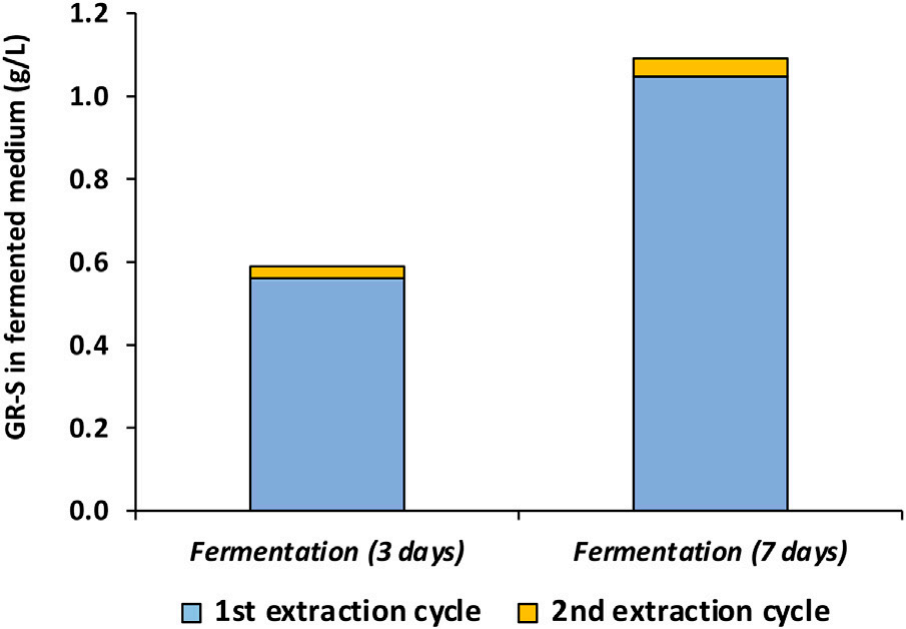
Content of GR-S (g/L), determined by UPLC-MS/MS, after the 3rd and 7th day of fermentation using 1 or 2 cycles of extraction with acidic ethanol.

Furthermore, to determine the maximum GR-S yield achievable per batch (Figure 4C), biomass underwent extraction under the most favorable conditions with ethanol:HCl at a 3:1 ratio v/v and for 120 min. To calculate total yield, two extraction cycles were performed after 3 or 7 days of fermentation as shown in the scheme of Supplementary Figure S5. Hence, Figure 5 shows the amount of GR-S detected respect to the fermented medium after 2 consecutive extraction cycles after 3 or 7 days of fermentation.

It was observed that the maximum concentration of GR-2 extractable in the first cycle was 0.561 g/L after 3 days of fermentation while this concentration increased to 1.047 g/L after 7 days of fermentation. Following a second extraction cycle, the Gramicidin concentration were only 0.028 g/L and 0.044 g/L of GR-2 after 3 and 7 days of fermentation, respectively. Therefore, when the most favorable conditions are used, 96% of GR-S can be extracted with a single cycle after 7 days of fermentation. In this case, considering that the dry weight of the crude extract was 1.164 g/L, the purity of the GR-S extract was 90%. It is evident that when the ratio of fermented medium to ethanol:HCl is 3:1 v/v, the yield ranged between 87.5% and 96.1%. Conversely, when the ratio of medium to ethanol:HCl was adjusted to 6:1 v/v, the yield notably decreased to 56.3%–56.7% (Figure 4C). Furthermore, upon comparing the UPLC-MS/MS analysis of this trial with the extraction data of ethanol:HCl provided in Table 3 for GR-S, a significant agreement is observed with a similarity index (determined by FTIR analysis) and purity (determined by UPLC- MS/MS) relative to commercial GR-S around 90.0% in both cases.

In comparison with the literature, Nakai et al. (2005) produced a Gramicidin extract from *B. brevis* submitting the microbial biomass to a solid-liquid extraction with ethanol at high temperature (95°C). The extraction process was followed by a purification step of the supernatant by means of a basic alumina column. Moreover, in this case, Gramicidin elution from the column was carried out with 85% ethanol together with the separation process consisting of various steps under severe extraction conditions. Under this scenario, the authors achieved 0.045 g/L up to 0.20 g/L after 24 h of fermentation quantifying the Gramicidin concentration in the extract using a spectrophotometric method measuring the absorbance of samples at 600 nm (Nakai et al., 2005).

Recently, Nesteruk and Syrov (2017) used a mutant able to produce a high concentration of GR-S (2 g/L) in a synthetic medium supplemented with 40% of lactic acid. This value is a little bit higher than the maximum value achieved in the current work (1.2–1.3 g/L), although the yield of crude Gramicidin expressed in gram of biomass (0.2 g_gramicidin_ s/g_biomass_) was in the range of that obtained from *A. aneurinilyticus*. However, since the mutant strain was used in the previous study (Nesteruk and Syrov, 2017), their Gramicidin extract can be hardly compared with the Gramicidin extract obtained from a wild strain of *A. aneurinilyticus* that was isolated from CSL, a naturally fermented secondary stream, and used in the present work. Nesteruk and Syrov (2017) have proposed a protocol involving several stages, where, in a first stage, the biomass is filtered, washed with deionized water, and dried in a spray dryer at 180°C. Moreover, in a second stage, the biomass is washed with acetone to remove impurities and dried under vacuum at 45°C. Following, Gramicidin S is extracted with ethanol, the pH is adjusted to 3.0–4.0 using diluted HCl, and the biomass is dried under vacuum. Finally, the biomass is dissolved, decolorized with activated carbon, filtered, and rinsed with ethanol. On the other hand, Berditsch et al. (2007) used pre-extraction treatment of biomass using a saline solution at 80°C to facilitate Gramicidin release from the cells. According to another study, the highest values (0.231–0.301 g/L) of GR-S were obtained using a complex synthetic medium supplemented by certain amino acids and vitamins while it was produced only 0.109 g/L of GR-S using a basal minimal medium mainly consisting of glycerol, phosphate salts, ammonium sulphate, tris-HCl, etc. (Berditsch et al., 2015). The described process (Berditsch et al., 2015) yielded an amount of Gramicidin extract lower than that reported in the current work using a commercial basal fermented medium and employing a simple extraction process with ethanol as an extractant agent.

Moreover, to assess the antimicrobial potency of the extract with the highest yield (GR-2), its effectiveness was tested using a disk diffusion method. Inhibition zones around the disks impregnated with both GR-2 and commercial GR-S were observed, particularly in relation to the growth of the antagonist microorganism *B. licheniformis* (Supplementary Figure S6). This observation highlights the antimicrobial efficacy of GR-2.

In terms of economic analysis, focusing on the recovery of solvent as critical step, the Gramicidin extraction suggests that a process combining extraction with a vacuum distillation system for recovery of ethanol is the optimal choice. This protocol is endorsed as a best available technique by The Center for Sustainable Consumption and Production (SCP/RAC) of Spain (CPRAC, 2024) operating within the framework of the Mediterranean Action Plan (MAP) under the United Nations Environment Program (UNEP). Drawing from the example for solvent recovery (file N° 84) provided on the web site (CPRAC, 2024), the cost of implementing a vacuum distillation system for ethanol recovery during the Gramicidin downstream process was calculated. This model entails the recovery of 56,221 kg of solvent per year while the total losses amounted to 6,247 kg per year. Thus, the total amount of solvent (62,468 kg) treated in 1 year will be equivalent to 79,173 L of ethanol considering that ethanol density is equal to 789 kg/m³. The operational cost is estimated at €11,713 per year. Based on these figures and considering a theoretical period of fermentation and extraction equal to 172 h (7,912 h per year), it is projected that 46 batches could be processed annually. Consequently, each batch should yield 1,721 L of ethanol and produce 5.40 kg/batch of GR-S with the purity of 90%. This translates to an annual production of 248 kg of GR-S extract. The operational cost of the vacuum distillation system should ideally be three times lower (€47 per kg of GR-S) in comparison with the process proposed by Berditsch et al. (2015) (€141 per kg of GR-S). This comparison is drawn considering the fact that Berditsch et al. (2015) proposed the ratio of fermented media to extractant (1:1), which is lower than the 3:1 ratio proposed in the current work. Additionally, the GR-S amounts relative to the fermented medium proposed by Berditsch et al. (2015) (1.146 g/L) closely resemble the GR-S amounts obtained in the current work (1.047 g/L), while the authors generated only 0.109 g/L of GR-S when a basal medium was used. It's worth noting that in advance of GR-S extraction Berditsch et al. (2015) utilized a pretreatment process consisting of cells freezing at 20°C and pre-extraction in 150 mM NaCl and 20 mM HCl at 80°C for 15 min. This could potentially inflate the cost of GR-S extraction in comparison with the extraction process outlined in the present work. Furthermore, Berditsch et al. (2015) achieved comparable GR-S fermentation yields using a nutrient-rich medium named G4/4 that contains several amino acids (10 g/L AL-arginine; 1 g/L L-phenylalanine, 0.5 g/L L-methionine, 1.3 g/L L-histidine) in addition to salts and 10 g/L of glycerol. However, the cost of fermentation significantly increases due to increases in the cost of the fermented medium. For instance, the presence of amino acids alone in the fermented medium can potentially increase its price up to 37%–59% (Morais and Suárez, 2016).

In summary, our results demonstrated that Gramicidin S can be extracted using a single step with acidic ethanol, which represents a significant advancement in producing more sustainable Gramicidin S. Traditional methods reported in the literature involve multiple stages with higher energy and reagent consumption, often conducted at elevated temperatures. Additionally, this study showed that ethanol in an acidic environment (0.2 N HCl) was more effective than using ethanol alone or a PBS buffer solution for extracting Gramicidin S from *A. aneurinilyticus* biomass under mild conditions. Moreover, it was observed that when acidic ethanol was employed, the extraction time between 30 and 120 min had a negligible effect, being of critical importance the ratio of extractant. The extraction yields achieved with ethanol:HCl as an extractant agent in the present study are similar or even superior to those reported in the literature that described Gramicidin extraction with ethanol. Our results highlight the advantages of the methodology selected in this work in comparison with others, such as the lower number of downstream steps, lower extraction time, lower reagent consumption and no use of mutant strains. MALDI-TOF-MS spectra also revealed that the selected Gramicidin extract (GR-2) presents intensive signals at 1,141 m*/z* and 1,163 m*/z*, like commercial GR-S, discharging the presence of GR-A as no signals were detected around 1881–1909 m*/z*. Therefore, it can be established that a simple extraction process with acidic ethanol at room temperature and under reduced extraction time (30 min–120 min) is adequate to produce a GR-S of high purity from *A. aneurinilyticus*.

## Data Availability

The raw data supporting the conclusions of this article will be made available by the authors, without undue reservation.

## Abbreviations

GR-S, Gramicidin S; GR-A, Gramicidin A; TFA, trifluoroacetic acid; FTIR, Fourier Transform Infrared Spectroscopy; MALDI-TOF-MS, Matrix Assisted Laser Desorption/Ionization Time-of- Flight Mass Spectrometry; ESI-MS, Electrospray Ionization Mass Spectrometry; UPLC, Ultra Performance Liquid Chromatography; UPLC-MS/MS, Ultra Performance Liquid Chromatography Masses/Masses; DSMZ, German Collection of Microorganisms and Cell Cultures; CSL, corn steep liquor; TSB, Tryptic Soy Broth; TSA, Tryptic Soy Agar; PBS; phosphate buffer solution; GR-1, GR extracts of ethanol; GR-2, GR extracts of Ethanol:HCl; GR-3, PBS GR extract; ATR-FTIR, Total reflectance FTIR, r_P_, Pearson correlation factor; r_S_, Spearman correlation factor; P, purification index; P_P_, P index based on r_P_; P_S_, P index based on r_s_; SCP/RAC, Center for Sustainable Consumption and Production; MAP, Mediterranean Action Plan; UNEP, United Nations Environment Program.

## References

[B1] Abmole (2024). AbMole BioScience. Available at: https://www.abmole.com (Accessed July 18, 2024).

[B2] Abraham T. Prenner E. J. Lewis R. N. A. H. Mant C. T. Keller S. Hodges R. S. (2014). Structure – activity relationships of the antimicrobial peptide gramicidin S and its analogs: aqueous solubility, self-association, conformation, antimicrobial activity and interaction with model lipid membranes. Biochim. Biophys. Acta 1838, 1420–1429. 10.1016/j.bbamem.2013.12.019 24388950

[B3] Adams E. Schepers R. Gathu L. W. Kibaya R. Roets E. Hoogmartens J. (1997). Liquid chromatographic analysis of a formulation containing polymyxin, gramicidin and neomycin. J. Pharm. Biomed. Anal. 15, 505–511. 10.1016/s0731-7085(96)01881-x 8953494

[B4] Alenezi F. N. Rekik I. Bouket A. C. Luptakova L. Weitz H. J. Rateb M. E. (2017). Increased biological activity of *Aneurinibacillus migulanus* strains correlates with the production of new gramicidin secondary metabolites. Front. Microbiol. 8, 517. 10.3389/fmicb.2017.00517 28439259 PMC5383652

[B5] Berditsch M. Afonin S. Steineker A. Orel N. Jakovkin I. Weber C. (2015). Fermentation and cost-effective 13C/15N labeling of the nonribosomal peptide gramicidin S for nuclear magnetic resonance structure analysis. Appl. Environ. Microbiol. 81, 3593–3603. 10.1128/AEM.00229-15 25795666 PMC4421037

[B6] Berditsch M. Afonin S. Ulrich A. S. (2007). The ability of *Aneurinibacillus migulanus* (Bacillus brevis) to produce the antibiotic gramicidin S is correlated with phenotype variation. Appl. Environ. Microbiol. 73, 6620–6628. 10.1128/AEM.00881-07 17720841 PMC2075075

[B7] Berditsch M. Trapp M. Afonin S. Weber C. Misiewicz J. Turkson J. (2017). Antimicrobial peptide gramicidin S is accumulated in granules of producer cells for storage of bacterial phosphagens. Sci. Rep. 7, 44324. 10.1038/srep44324 28295017 PMC5353757

[B8] Bérdy J. (2005). Bioactive microbial metabolites. A personal view. J. Antibiot. (Tokyo) 58, 1–26. 10.1038/ja.2005.1 15813176

[B9] Bruckner H. Westhauser T. Godel H. (1995). Liquid chromatographic determination of D-and L-amino acids by derivatization with o-phthaldialdehyde and N-isobutyryl-L-cysteine Applications with reference to the analysis of peptidic antibiotics, toxins, drugs and pharmaceutically used amino acids. J. Chromatogr. A 711, 201–215. 10.1016/0021-9673(95)00158-j 7496491

[B10] Bustos G. Arcos U. Vecino X. Cruz J. M. Moldes A. B. (2018). Recycled Lactobacillus pentosus biomass can regenerate biosurfactants after various fermentative and extractive cycles. Biochem. Eng. J. 132, 191–195. 10.1016/j.bej.2018.01.021

[B11] Cabral-Romero C. García-Cuellar C. M. Hernandez-Delgadillo R. Sánchez-Pérez Y. Meester I. Solís-Soto J. M. (2021). Synergistic antitumor activity of gramicidin/lipophilic bismuth nanoparticles (BisBAL NPs) on human cervical tumor cells. Front. Nanotechnol. 3. 10.3389/fnano.2021.633604

[B12] CPRAC (2024). Regional activity centre for sustainable consumption and production. Available at: http://www.cprac.org (Accessed April 30, 2024).

[B13] David J. M. Rajasekaran A. K. (2015). Gramicidin A: a new mission for an old antibiotic. J. Kidney Cancer VHL 2, 15–24. 10.15586/jkcvhl.2015.21 28326255 PMC5345515

[B14] Dubos R. J. Hotchkiss R. D. (1941). The production of bactericidal substances by aerobic sporulating Bacilli. J. Exp. Med. 73, 629–640. 10.1084/jem.73.5.629 19871101 PMC2135154

[B15] Erve J. C. L. Gu M. Wang Y. DeMaio W. Talaat R. E. (2009). Spectral accuracy of molecular ions in an LTQ/Orbitrap mass spectrometer and implications for elemental composition determination. J. Am. Soc. Mass Spectrom. 20, 2058–2069. 10.1016/j.jasms.2009.07.014 19716315

[B16] Fang A. Pierson D. L. Mishra S. K. Koenig D. W. Demain A. L. (1997). Gramicidin S production by Bacillus brevis in simulated microgravity. Curr. Microbiol. 34, 199–204. 10.1007/s002849900168 9058537

[B17] Freitas de Oliveira D. W. Lima França Í. W. Nogueira Félix A. K. Lima Martins J. J. Aparecida Giro M. E. Melo V. M. M. (2013). Kinetic study of biosurfactant production by Bacillus subtilis LAMI005 grown in clarified cashew apple juice. Colloids Surf. B Biointerfaces 101, 34–43. 10.1016/j.colsurfb.2012.06.011 22796769

[B18] Gause G. F. Brazhnikova M. G. (1944). Gramicidin s and its use in the treatment of infected wounds. Nature 703, 703. 10.1038/154703a0

[B19] Goto K. Fujita R. Kato Y. Asahara M. Yokota A. (2004). Reclassification of *Brevibacillus brevis* strains NCIMB 13288 and DSM 6472 (=NRRL NRS-887) as Aneurinibacillus danicus sp. nov. and Brevibacillus limnophilus sp. nov. Int. J. Syst. Evol. Microbiol. 54, 419–427. 10.1099/ijs.0.02906-0 15023954

[B20] Henschel H. Andersson A. T. Jespers W. Mehdi Ghahremanpour M. Van Der Spoel D. (2020). Theoretical infrared spectra: quantitative similarity measures and force fields. J. Chem. Theory Comput. 16, 3307–3315. 10.1021/acs.jctc.0c00126 32271575 PMC7304875

[B21] Hotchkiss R. D. Dubos R. J. (1941). The isolation of bactericidal substances from cultures of Bacillus brevis. J. Biol. Chem. 141, 155–162. 10.1016/s0021-9258(18)72830-5

[B22] Kamli M. R. Alzahrani N. A. Y. Hajrah N. H. Sabir J. S. M. Malik A. (2021). Genome‐driven discovery of enzymes with industrial implications from the genus Aneurinibacillus. Microorganisms 9, 499. 10.3390/microorganisms9030499 33652876 PMC7996765

[B23] Kelkar D. A. Chattopadhyay A. (2007). The gramicidin ion channel: a model membrane protein. Biochim. Biophys. Acta Biomembr. 1768, 2011–2025. 10.1016/j.bbamem.2007.05.011 17572379

[B24] Kratzschmar J. Krause M. Marahiel M. A. (1989). Gramicidin S biosynthesis operon containing the structural genes grsA and grsB Has an open reading frame encoding a protein homologous to fatty acid thioesterases. J. Bacteriol. 171, 5422–5429. 10.1128/jb.171.10.5422-5429.1989 2477357 PMC210379

[B25] López-Prieto A. Rodríguez-López L. Rincón-Fontán M. Cruz J. M. Moldes A. B. (2021). Characterization of extracellular and cell bound biosurfactants produced by Aneurinibacillus aneurinilyticus isolated from commercial corn steep liquor. Microbiol. Res. 242, 126614. 10.1016/j.micres.2020.126614 33045681

[B26] Maione S. del Valle L. J. Pérez-Madrigal M. M. Cativiela C. Puiggalí J. Alemán C. (2016). Electrospray loading and release of hydrophobic gramicidin in polyester microparticles. RSC Adv. 6, 73045–73055. 10.1039/c6ra11056h

[B27] Mariotti F. Tomé D. D. Patureau Mirand P. (2008). Converting nitrogen into protein-beyond 6.25 and Jones’ factors. Crit. Rev. Food Sci. Nutr. 48, 177–184. 10.1080/10408390701279749 18274971

[B28] MedChemExpress (2024). Master of bioactive molecules. Available at: https://www.medchemexpress.com (Accessed July 18, 2024).

[B29] Morais V. Suárez N. (2016). Economic evaluation of *Streptococcus pneumoniae* culture media. Am. J. Biochem. Biotechnol. 12, 133–138. 10.3844/ajbbsp.2016.133.138

[B30] Nakai T. Yamauchi D. Kubota K. (2005). Enhancement of linear gramicidin expression from *Bacillus brevis* ATCC 8185 by casein peptide. Biosci. Biotechnol. Biochem. 69, 700–704. 10.1271/bbb.69.700 15849407

[B31] Nesteruk V. V. Syrov K. K. (2017). Method for producing and purification of gramicidin S. European patent application EP3660141A1.

[B32] Orwa J. A. Govaerts C. Roets E. Schepdael A. V. Hoogmartens J. (2001). Liquid chromatography of gramicidin. Chromatographia 53, 17–21. 10.1007/BF02492421

[B33] Pavithrra G. Rajasekaran R. (2020). Gramicidin peptide to combat antibiotic resistance: a review. Int. J. Pept. Res. Ther. 26, 191–199. 10.1007/s10989-019-09828-0

[B34] Pfukwa N. B. C. Rautenbach M. Hunt N. T. Olaoye O. O. Kumar V. Parker A. W. (2023). Temperature-induced effects on the structure of gramicidin S. J. Phys. Chem. B 127, 3774–3786. 10.1021/acs.jpcb.2c06115 37125750

[B35] Pittenauer E. Zehl M. Belgacem O. Raptakis E. Mistrik R. Allmaier G. (2006). Comparison of CID spectra of singly charged polypeptide antibiotic precursor ions obtained by positive-ion vacuum MALDI IT/RTOF and TOF/RTOF, AP-MALDI-IT and ESI-IT mass spectrometry. J. Mass Spectrom. 41, 421–447. 10.1002/jms.1032 16604520

[B36] Prat D. Hayler J. Wells A. (2014). A survey of solvent selection guides. Green Chem. 16, 4546–4551. 10.1039/c4gc01149j

[B37] Rincón-Fontán M. Rodríguez-López L. Vecino X. Cruz J. M. Moldes A. B. (2017). Influence of micelle formation on the adsorption capacity of a biosurfactant extracted from corn on dyed hair. RSC Adv. 7, 16444–16452. 10.1039/c7ra01351e

[B38] Schuster C. Schmitt A. (2018). Efficacy of a bacterial preparation of *Aneurinibacillus migulanus* against downy mildew of cucumber (Pseudoperonospora cubensis). Eur. J. Plant Pathol. 151, 439–450. 10.1007/s10658-017-1385-4

[B39] Shida O. Takagi H. Kadowaki K. Komagata K. (1996). Proposal for two new genera, Brevibacillus gen. nov. and Aneurinibacillus gen. nov. Int. J. Syst. Bacteriol. 4, 939–946. 10.1099/00207713-46-4-939 8863420

[B40] Solovskij M. Panarin E. (1999). Polymer water-soluble derivatives of polypeptide antibiotic, gramicidin-S based on reactive copolymers of *N*-(2-hydroxypropyl) methacrylamide. J. Control Release 58, 1–8. 10.1016/s0168-3659(98)00126-6 10021484

[B41] Swierstra J. Kapoerchan V. Knijnenburg A. van Belkum A. Overhand M. (2016). Structure, toxicity and antibiotic activity of gramicidin S and derivatives. Eur. J. Clin. Microbiol. Infect. Dis. 35, 763–769. 10.1007/s10096-016-2595-y 26886453 PMC4840228

[B42] Torres-Sangiao E. Leal Rodriguez C. García‐Riestra C. (2021). Application and perspectives of MALDI–TOF mass spectrometry in clinical microbiology laboratories. Microorganisms 9, 1539. 10.3390/microorganisms9071539 34361974 PMC8307939

[B43] Valenti L. E. Paci M. B. De Pauli C. P. Giacomelli C. E. (2011). Infrared study of trifluoroacetic acid unpurified synthetic peptides in aqueous solution: trifluoroacetic acid removal and band assignment. Anal. Biochem. 410, 118–123. 10.1016/j.ab.2010.11.006 21078284

[B44] Vecino X. Barbosa-Pereira L. Devesa-Rey R. Cruz J. M. Moldes A. B. (2015). Optimization of extraction conditions and fatty acid characterization of Lactobacillus pentosus cell-bound biosurfactant/bioemulsifier. J. Sci. Food Agric. 95, 313–320. 10.1002/jsfa.6720 24798413

[B45] Wang F. Qin L. Pace C. J. Wong P. Malonis R. Gao J. (2012). Solubilized Gramicidin A as potential systemic antibiotics. ChemBioChem 13, 51–55. 10.1002/cbic.201100671 22113881

[B46] Wenzel M. Rautenbach M. Vosloo J. A. Siersma T. Aisenbrey C. H. M. Zaitseva E. (2018). The multifaceted antibacterial mechanisms of the pioneering peptide antibiotics tyrocidine and gramicidin S. mBio 9, e00802-18. 10.1128/mBio.00802-18 30301848 PMC6178620

[B47] Xue Y. W. Itoh H. Dan S. Inoue M. (2022). Gramicidin A accumulates in mitochondria, reduces ATP levels, induces mitophagy, and inhibits cancer cell growth. Chem. Sci. 13, 7482–7491. 10.1039/d2sc02024f 35872830 PMC9241976

