# Supplementary material for: Efficient one step extraction process of Gramicidin S from Aneurinibacillus aneurinilyticus biomass
Source: Front Bioeng Biotechnol. 2024 Aug 29;12:1452796. doi: 10.3389/fbioe.2024.1452796 (PMC11390548; doi:10.3389/fbioe.2024.1452796)
Supplement: Supplementary file 1 [file DataSheet1.docx]

**EFFICIENT ONE STEP EXTRACTION PROCESS OF GRAMICIDIN S FROM *Aneurinibacillus aneurinilyticus* BIOMASS**

Ksenia Lvova^1^, Xanel Vecino^1^, Benita Pérez-Cid^2^, Ana B. Moldes^1^* and José M. Cruz^1^

^1^Chemical Engineering Department, School of Industrial Engineering -CINTECX, University of Vigo, Campus As Lagoas-Marcosende, 36310 Vigo, Spain.

^2^Department of Analytical and Food Chemistry, Faculty of Chemistry-CINTECX, University of Vigo, Campus As Lagoas-Marcosende, 36310 Vigo, Spain.

*Correspondence: [amoldes@uvigo.gal](mailto:amoldes@uvigo.gal)

**Table S1**. Summary of Gramicidin extraction methods from different *Bacillus* strains.

| Microorganism  (strain) | Microbial source | GR form^1^ | Extraction method | Reference |
| --- | --- | --- | --- | --- |
| *Bacillus brevis* | Soil | D | Cells acidification;  Extraction with acetone and ether;  Crystallization by acetone | [19] |
| *Bacillus brevis*  (Nagano) | No data | S | Extraction with ethanol and HCl at ahigh temperature | [12] |
| *Bacillus brevis* | Soil | D | Multi-step extraction process | [20] |
| *A. migulanus*  (VKPM B-10212) | Mutant created in llaboratory | S | Ultrafiltration, washing and drying of cells;  Washing by acetone; Extraction with ethanol and HCl repeatedly;  Biomass decolorization by carbon, filtration and washing with ethanol;  Clarification with NaCl;  Crystallization | [21] |
| *A. migulanus*  (ATCC 9999^T^ = DSM 2895^T^; DSM 5668;  DSM 5759) | German Collection of Microorganisms and Cell Cultures (DSMZ) | S | Cells freezing;  Preextraction by NaCl and HCl;  Extraction with ethanol | [22,23] |

^1^Mixtures of linear Gramicidin A, B, C are denoted as Gramicidin D


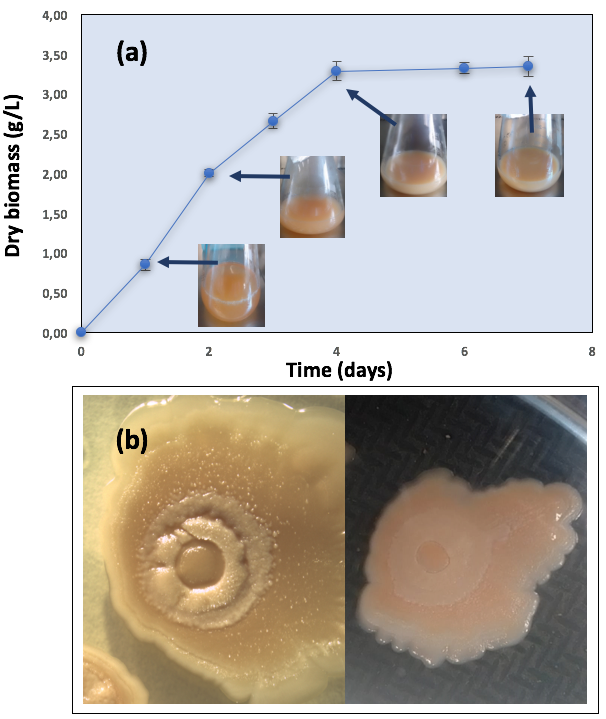


**Figure S1**. (a) Production of dry microbial biomass (g/L) in the synthetic fermentation medium; (b) Pictures of *A. aneurinilyticus* colonies isolated on agar medium from TSB.


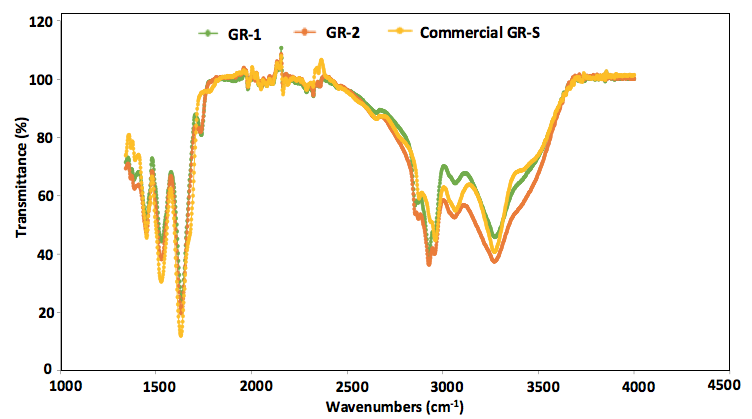


**Figure S2.** Comparison of FTIR spectra of Commercial GR-S with GR-1 and GR-2 between 1250-4000 cm^-1^.


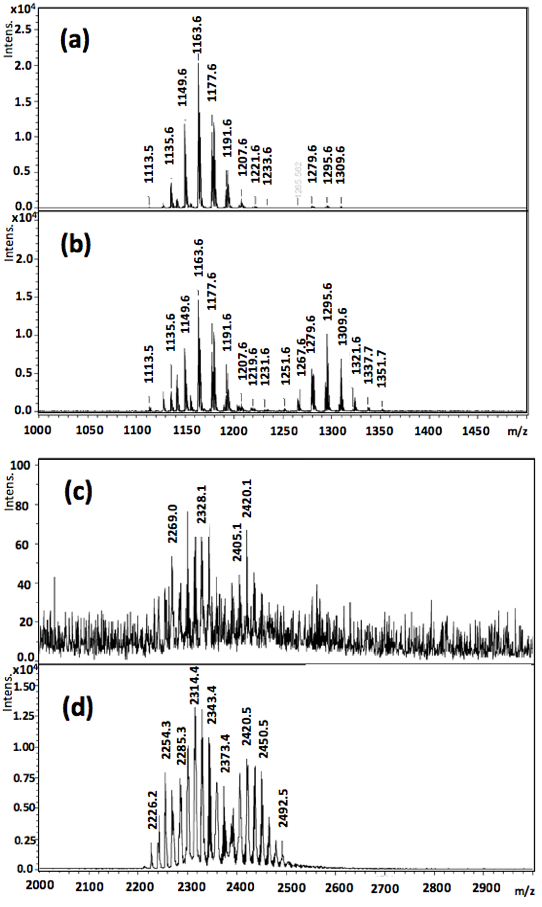


**Figure S3.** MALDI-TOF MS spectra of Gramicidin extract GR-2 in acidic ethanol obtained at a) room temperature, masses between 1000 and 1500 m/z; b) 40 ºC, masses between 1000 and 1500 m/z; c) room temperature, masses between 2000 and 3000 m/z; d) 40 ºC, masses between 2000 and 3000 m/z.


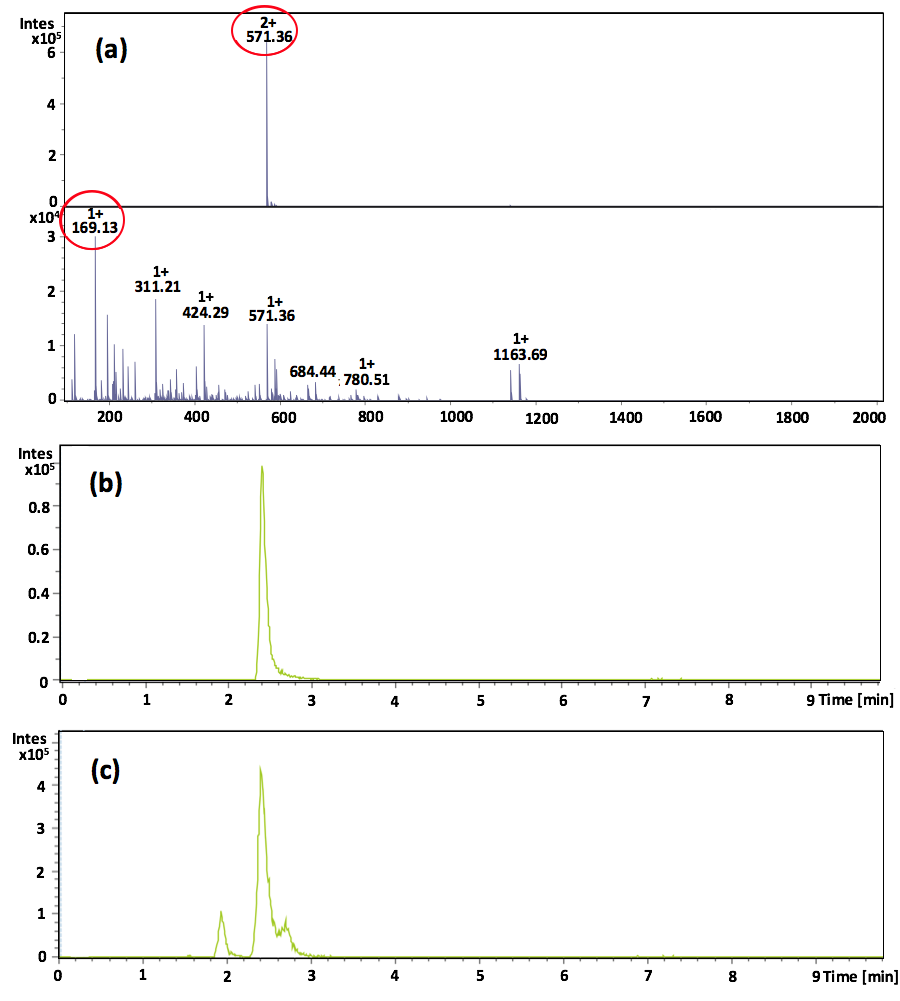


**Figure S4.** (a) Scan mass and MS/MS spectra of commercial GR-S at a concentration of 5 mg/mL and extracted ion chromatogram (EIC) of: (b) commercial GR-S and (c) GR-2.


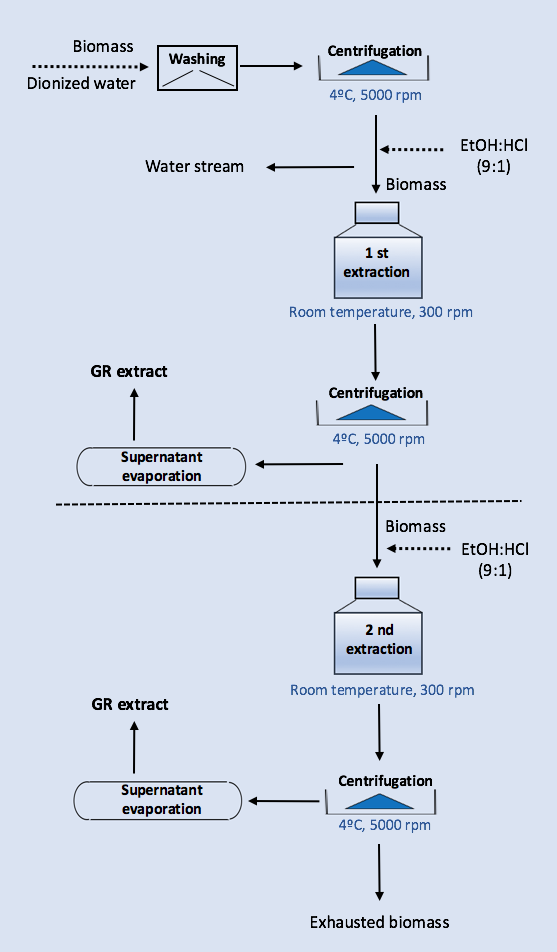


**Figure S5**. Scheme of two consecutive extraction cycles of Gramicidin from the microbial biomass.


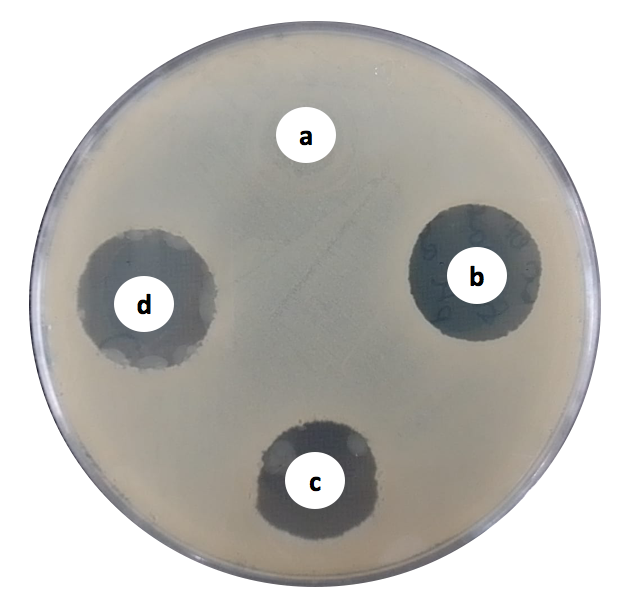


**Figure S6**. Zone of inhibition of *Bacillus* *licheniformis* on TSA: (a) Control without Gramicidin (b) Commercial GR-S at 1 g/L; (c) GR-2 at 1 g/L; (d) GR-2 at 3 g/L.
